# Changes in Sulfur Metabolism in Mouse Brains following Radon Inhalation

**DOI:** 10.3390/ijerph191710750

**Published:** 2022-08-29

**Authors:** Norie Kanzaki, Akihiro Sakoda, Takahiro Kataoka, Lue Sun, Hiroshi Tanaka, Iwao Ohtsu, Kiyonori Yamaoka

**Affiliations:** 1 Ningyo-Toge Environmental Engineering Center, Japan Atomic Energy Agency, 1550 Kamisaibara, Kagamino-cho, Tomata-gun, Okayama 708-0698, Japan; 2 Faculty of Health Sciences, Okayama University, 5-1 Shikata-cho 2-chome, Kita-ku, Okayama 700-8558, Japan; 3 Health and Medical Research Institute, Department of Life Science and Biotechnology, National Institute of Advanced Industrial Science and Technology (AIST), Central 6, 1-1-1 Higashi, Tsukuba, Ibaraki 305-8566, Japan; 4 Faculty of Life and Environmental Sciences, University of Tsukuba, 1-1-1 Tennodai, Tsukuba 305-8577, Japan

**Keywords:** radon, sulfur metabolism, glutathione

## Abstract

Therapy using hot springs, including the high-level radioactive gas “radon”, is traditionally conducted as an alternative treatment for various diseases. Oxidative-stress-related diseases are inhibited by the enhancement of antioxidative functions following radon inhalation. We have reported that radon inhalation increased the level of anti-oxidants, such as glutathione (G-SH), in the brain and had a protective antioxidative effect against transient global cerebral ischemic injury. However, no studies have yet revealed the changes in G-SH associated substances after radon inhalation. In this study, we comprehensively analyzed several metabolites, focusing on G-SH. Mice were exposed to radon at concentrations of 200, 2000, or 20,000 Bq/m^3^ for 1, 3, or 10 days. We detected 27 metabolites in the mouse brains. The result showed that the L-methionine levels increased, whereas the levels of urea, glutathione, and sulfite ion decreased under any condition. Although the ratio of G-SH to oxidized glutathione (GS-SG) decreased, glutathione monosulfide (G-S-SH) and cysteine monosulfide (Cys-S-SH) increased after radon inhalation. G-S-SH and Cys-S-SH can produce a biological defense against the imbalance of the redox state at very low-dose irradiation following radon inhalation because they are strong scavengers of reactive oxygen species. Additionally, we performed an overall assessment of high-dimensional data and showed some specific characteristics. We showed the changes in metabolites after radon inhalation using partial least squares-discriminant analysis and self-organizing maps. The results showed the health effects of radon, especially the state of sulfur-related metabolites in mouse brains under the exposure conditions for radon therapy.

## 1. Introduction

Radon, a radioactive gas, is ubiquitous in the atmosphere. Exposure to radon and its decay products results in an increased risk of lung cancer [1,2]. In contrast, therapy using radon hot springs is known as an alternative treatment for bronchial asthma or rheumatic diseases [3,4]. Although determining the risks associated with radon therapy is not easy, because of many unknown factors such as natural backgrounds, smoking behaviors, and health conditions, the risks and benefits of radon therapy should be considered in detail [5]. It can be inferred that the production of a modest amount of free radical and reactive oxygen species (ROS) is essential for homeostatic regulation of redox signaling, although excessive amounts of these produced by irradiation are toxic. Therefore, understanding the oxidative stress response following radon inhalation is important.

Glutathione (G-SH) is an antioxidant with the thiol group (-SH). G-SH can be converted to oxidized glutathione (GS-SG) through glutathione peroxidase and converted back to G-SH by glutathione reductase. The GS-SG/G-SH ratio is an oxidative stress parameter [6]. Recent investigations have demonstrated that G-[S]*_n_*-SH (*n* > 1) can be formed by glutathione reductase-mediated reduction of GS-[S]*_n_*-SG and is a strong ROS-scavenger [7]. In cellular protection, substances with sulfur atoms bound to thiol (-[S]*_n_*-SH) have more protective effects on cells than those with thiol groups (-SH), and the same applies to cysteine (Cys-SH) [8]. The regulation of various G-SH- and Cys-SH-related substances is important for revealing the biological reaction against oxidative stress.

We previously reported that radon inhalation inhibited oxidative stress-related disease because of increased antioxidative function [9,10,11,12,13]. Although a consistent mechanism has not been clarified because of limited analytical indicators, previous studies have suggested some biological defense mechanisms against radon inhalation [14,15,16]. For example, a specific localization of G-SH in the cerebellum and hippocampus was observed, as well as an increase of G-SH in the brain evaluated after low-dose γ-irradiation (50 cGy) [17]. Our findings also suggest that radon inhalation (2000 Bq/m^3^ of radon for 24 h) increased the amount of antioxidants (such as G-SH) in the brain and had a protective antioxidative effect equivalent to 500 mg/kg of ascorbic acid treatment against transient global cerebral ischemic injury [10].

The primary goal of this study is to reveal changes in the sulfur metabolism, focusing on G-SH- and Cys-SH-related substances following radon inhalation in mouse brains. G-SH- or Cys-SH-related substances are deeply involved in oxidative stress and contribute to anti-inflammatory effects, but no studies have focused on changes in these substances following radon inhalation. In the present study, we considered the remarkable novel sulfur-containing compounds through metabolome analysis, which is a comprehensive analysis of metabolites. This study is essential for the discussion of oxidative stress response following radon inhalation.

## 2. Materials and Methods

### 2.1. Animals

Eight-week-old male BALB/c mice were purchased from CLEA Japan Inc. (Tokyo, Japan). The mice were kept under controlled room temperature (24 ± 2 °C) and 12 h light/dark cycles. The mice had free access to a standard diet (MF, Oriental Yeast Co., Ltd., Tokyo, Japan) and water. All of the experimental protocols in this study were approved by the Animal Experimentation Committee of Okayama University (OKU-2019158).

### 2.2. Radon Exposure and Sample Preparation

The mice were randomly divided into ten groups (six mice per group) and housed in a radon exposure system [18,19]. The radon concentrations in the breeding cages were measured using radon monitors (AlphaGUARD PQ2000 PRO, SAPHYMO, Frankfurt, Germany). In the control group, mice were kept in air without control of the radon concentration (around 20 Bq/m^3^) for 3 days. In the radon inhalation group, mice were exposed to radon at a concentration of about 200, 2000, or 20,000 Bq/m^3^ for 1, 3, or 10 days. Immediately after exposure, the mice were euthanized by carbon dioxide inhalation. Brain samples were quickly excised after blood sampling and stored at −80 °C until further analysis.

### 2.3. Sulfur Metabolomics Analysis

Sulfur metabolomics analysis was performed through the Sulfur Index Service (Euglena Co., Ltd., Tokyo, Japan). The sulfur-containing compounds in the samples were measured using a combination of liquid chromatography–tandem mass spectrometry (LC-MS/MS 8040, Shimadzu Corporation, Kyoto, Japan) and the thiol-specific derivatization method with monobromobimane, as in earlier reports [20,21,22,23,24]. The target metabolite levels were determined from the peak area by mass chromatography (i.e., relative amounts normalized with the peak area of the internal standard).

### 2.4. Statistical Analysis and Data Visualization Using Machine Learning

The mean ± standard deviation (SD) of each experimental group was calculated using the values normalized with the mean of the control group, and significant differences between the control group and the radon inhalation groups were analyzed by the one-way analysis of variance test and Dunnett’s test. A *p*-value < 0.05 was considered statistically significant. We used the Pearson product–moment correlation coefficient to assess correlations. In terms of the most correlated metabolites, single linear analysis was performed to identify the relationship between the metabolites and radon exposure (days or concentration). Partial least squares-discriminant analysis (PLS-DA) was performed for presenting an overall assessment of each metabolite. These analyses were executed with R (R Core Team (2019); R: a language and environment for statistical computing; the R Foundation for Statistical Computing, Vienna, Austria. https://www.R-project.org/. accessed on 29 July 2022). PLS-DA, which is a supervised version of principal component analysis, is used for the multivariate analysis method of diagnosis [25,26]. R^2^X, R^2^Y, Q^2^, and variable importance in projection (VIP) values indicate the accuracy of the PLS-DA model. In addition, we understood the whole context of the changes in metabolites using SOM_PAK (SOM Programming Team of the Helsinki University of Technology Laboratory of Computer and Information Science, Espoo, Finland), which is a tool for visualizing high-dimensional data. The output map of the self-organizing maps (SOM), which is a machine learning (ML) algorithm, is constructed by unsupervised learning of high-dimensional input numeric data [27]. Here, the number of learning, neighborhood radius, and learning rate were 100,000 times, 30 units, and 0.5, respectively. The map size was 30 × 20 units. We experimentally defined the parameters of SOM because a decisive approach for the definition of these has not yet been realized. A Z-score was used for these multivariate analyses.

## 3. Results

### 3.1. Changes in Metabolites of the Brains of Mice Exposed to Radon

We conducted metabolomics of the brains collected from the mice immediately after completing radon inhalation (200, 2000, or 20,000 Bq/m^3^ for 1, 3, or 10 days) to identify responsive sulfur-containing compounds to radon exposure. We analyzed 63 kinds of metabolites and detected 27 metabolites. Table 1 lists the 27 detected metabolites in the mouse brains.

**Figure 1:**
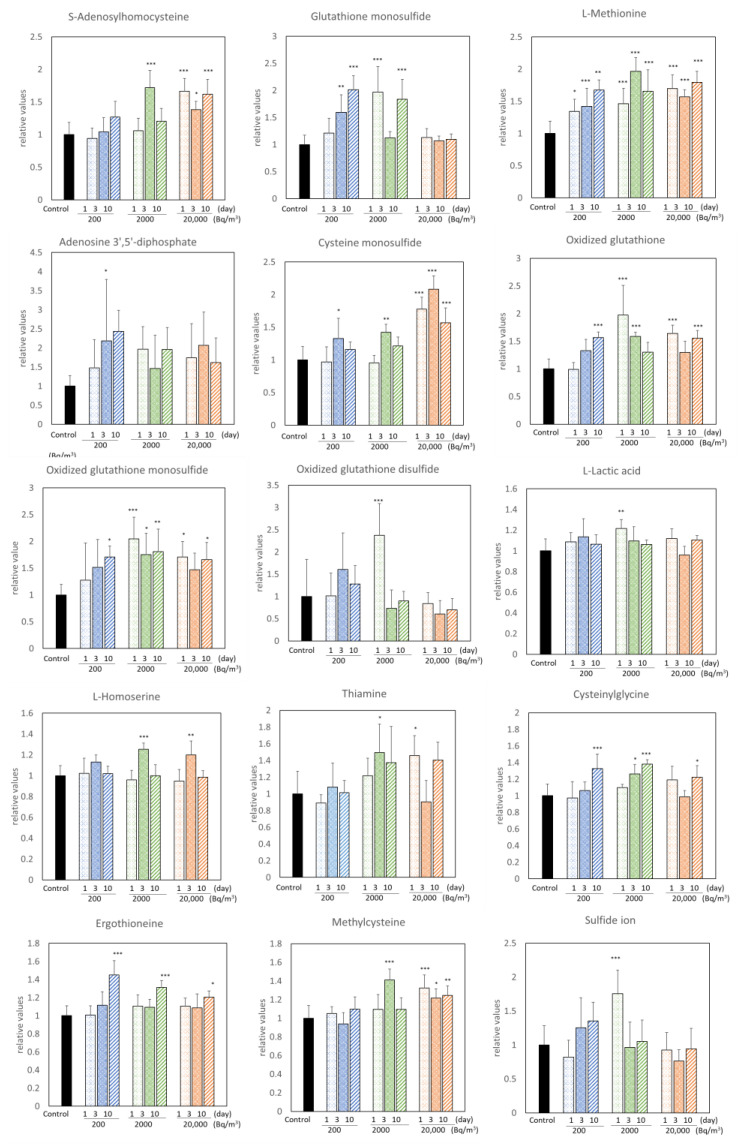
Metabolites significantly increased by radon inhalation. The number of mice per experimental point is six. All quantitative data are expressed as mean ± SD. * *p* < 0.05, ** *p* < 0.01, *** *p* < 0.001. vs. control.

**Figure 2:**
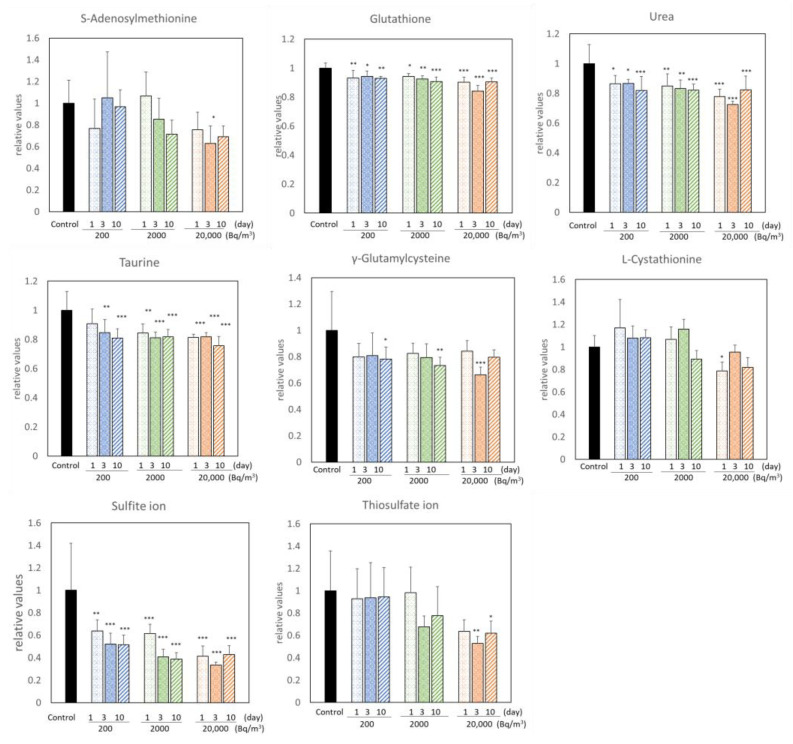
Metabolites significantly decreased by radon inhalation. The number of mice per experimental point is six. All quantitative data are presented as mean ± SD. * *p* < 0.05, ** *p* < 0.01, *** *p* < 0.001. vs. control.

**Figure 3:**
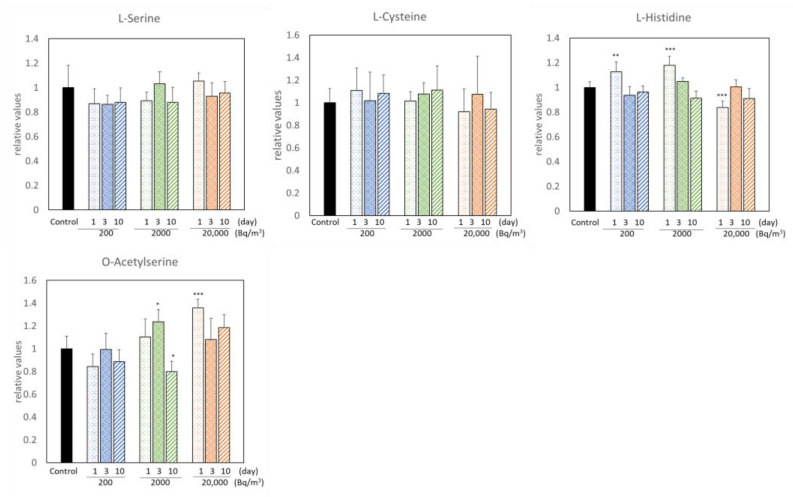
Metabolites with incoherent changes by radon inhalation. The number of mice per experimental point is six. All quantitative data are presented as mean ± SD. * *p* < 0.05, ** *p* < 0.01, *** *p* < 0.001. vs. control.

Radon inhalation increased the levels of 15 metabolites and decreased those of 8 metabolites (Figure 1 and Figure 2). Among these, radon inhalation especially increased the L-methionine level and decreased the urea, G-SH, and sulfite ion levels under any condition. The others had no notable features, depending on the radon concentration or radon inhalation time (Figure 3).

**Figure 4:**
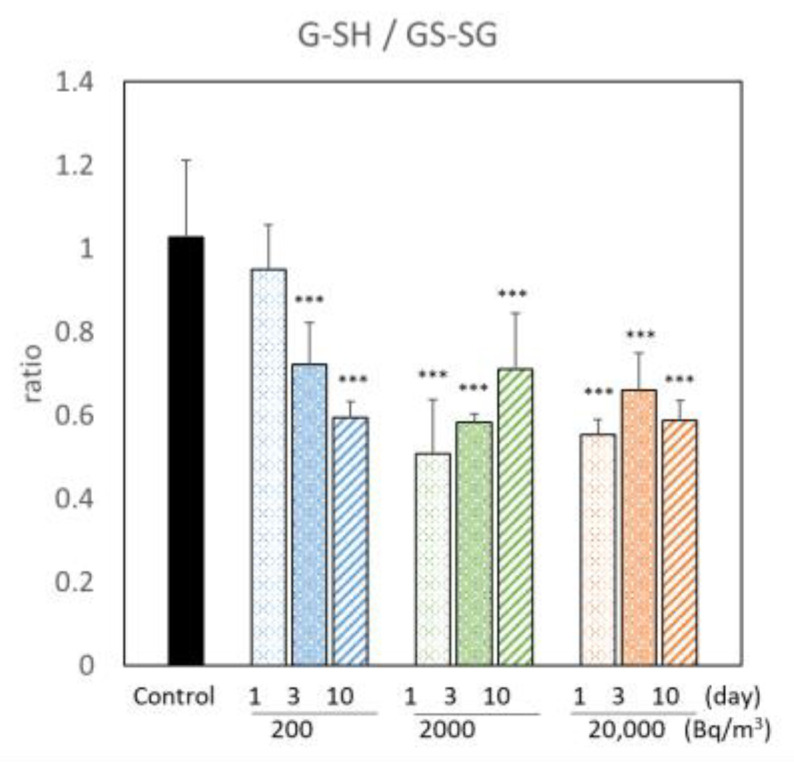
Changes in G-SH/GS-SG by radon inhalation. The number of mice per experimental point is six. All quantitative data are presented as mean ± SD. *** *p* < 0.001. vs. control.

We showed the ratio of GS-SG to G-SH, which is an oxidative stress parameter, to evaluate oxidative stress following radon inhalation (Figure 4). The ratio of G-SH to GS-SG was decreased by radon inhalation of more than 200 Bq/m^3^ for 3 days. This result suggests that radon inhalation causes a redox state imbalance in the mouse brains.

### 3.2. Analysis of the Relationship between Metabolites and Radon Inhalation Days or Radon Concentration

We found nine metabolites that were significantly correlated with radon inhalation days (0 (control), 1, 3, and 10) or radon concentration (0 (control), 200, 2000, and 20,000) (Table 2a,b). Cysteinylglycine and Ergothioneine were significantly correlated with radon inhalation days, whereas the others were significantly correlated with radon concentration. However, no metabolite was correlated with radon exposure amount (i.e., radon inhalation days × concentration).

**Figure 5:**
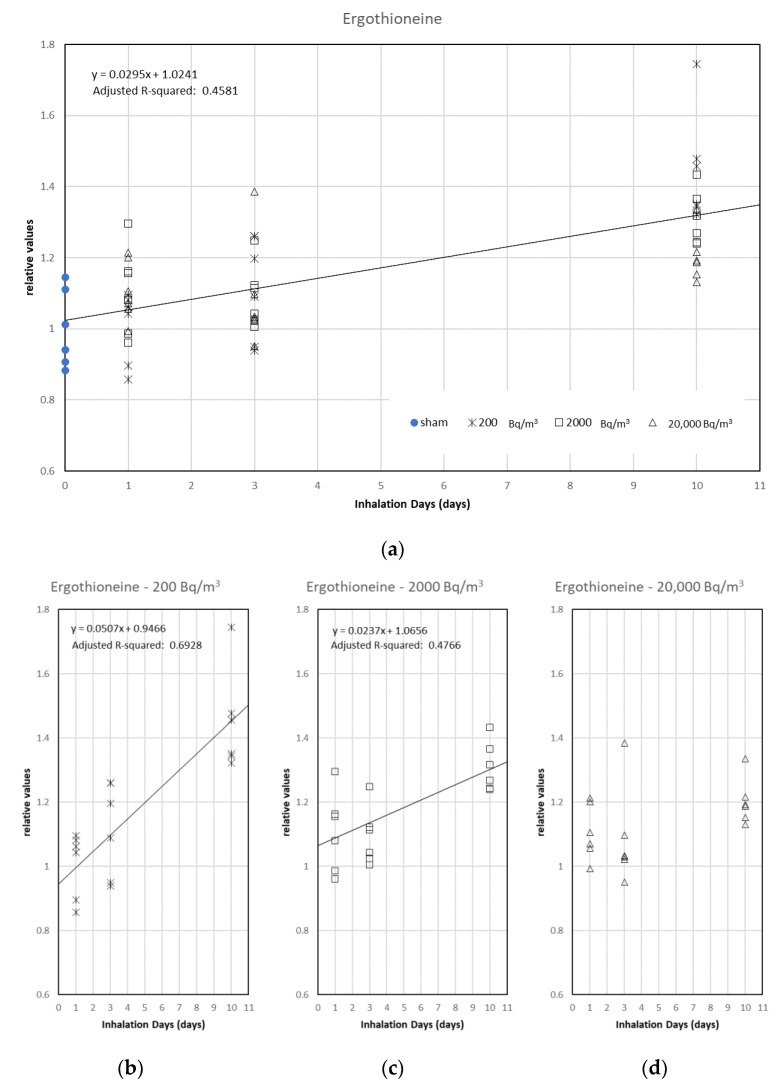
Scatter plots between inhalation days and Ergothioneine with a simple linear regression. The results are shown for (**a**) all groups, (**b**) 200 Bq/m^3^ groups, (**c**) 2000 Bq/m^3^ groups, and (**d**) 20,000 Bq/m^3^ groups (without a regression line because the results were not statistically significant).

**Figure 6:**
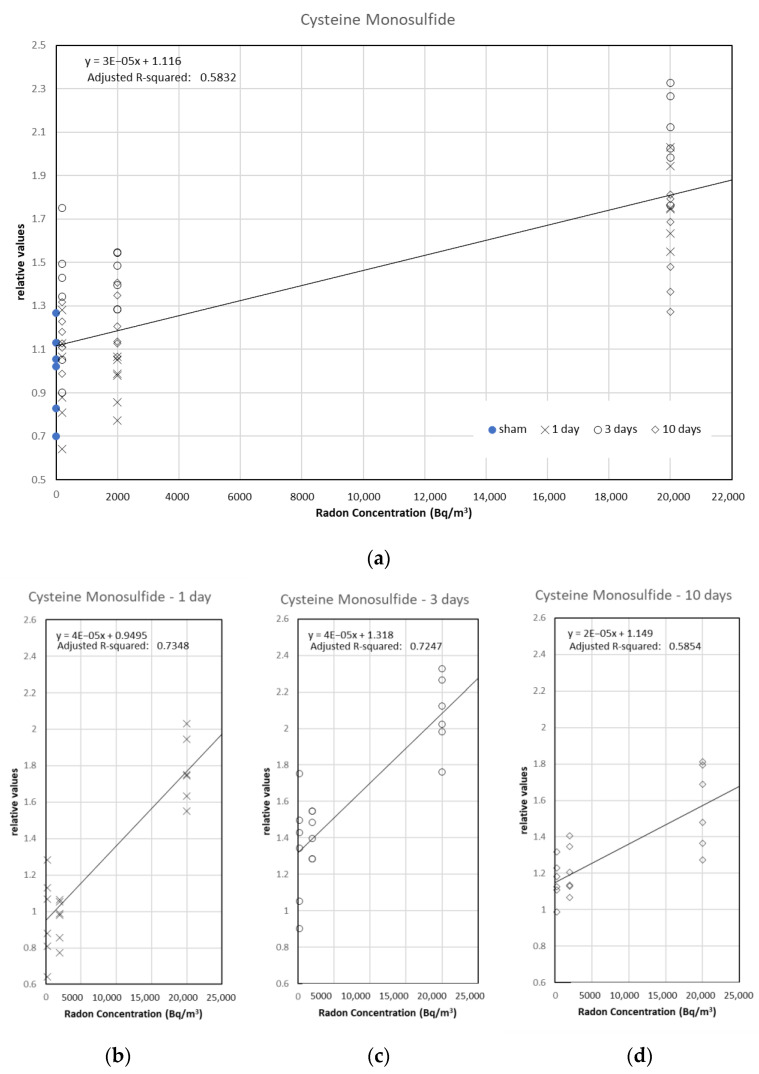
Scatter plots between radon concentration and Cys-S-SH with a simple linear regression. The results are shown for (**a**) all groups, (**b**) 1-day groups, (**c**) 3-day groups, and (**d**) 10-day groups.

Ergothioneine and Cys-S-SH were most correlated with radon inhalation days and radon concentration, respectively. Ergothioneine was marginally related to inhalation days in a simple regression model (coefficient of determination: 0.4581) (Figure 5a). The regression lines (*p* < 0.001) were calculated in 200 and 2000 Bq/m^3^ groups (Figure 5b,c). In the case of the 20,000 Bq/m^3^ group, the significant regression line was not found (*α* = 1.07934 [*p* < 0.001], *β* = 0.01174 [*p* = 0.114], and adjusted *R*-squared = 0.1019) (Figure 5d). Cys-S-SH was related to radon concentration in a simple regression model (coefficient of determination: 0.5832) (Figure 6a). The regression lines (*p* < 0.001) were calculated in the 1-, 3-, and 10-day groups (Figure 6b–d). These results suggest that the metabolites did not correlate with radon exposure when the mice were exposed to additional radon. Incidentally, we did not obtain a statistically meaningful result in the stepwise multiple regression analysis.

### 3.3. Visualization of the Effects of Radon Inhalation Using PLS-DA and ML

**Figure 7:**
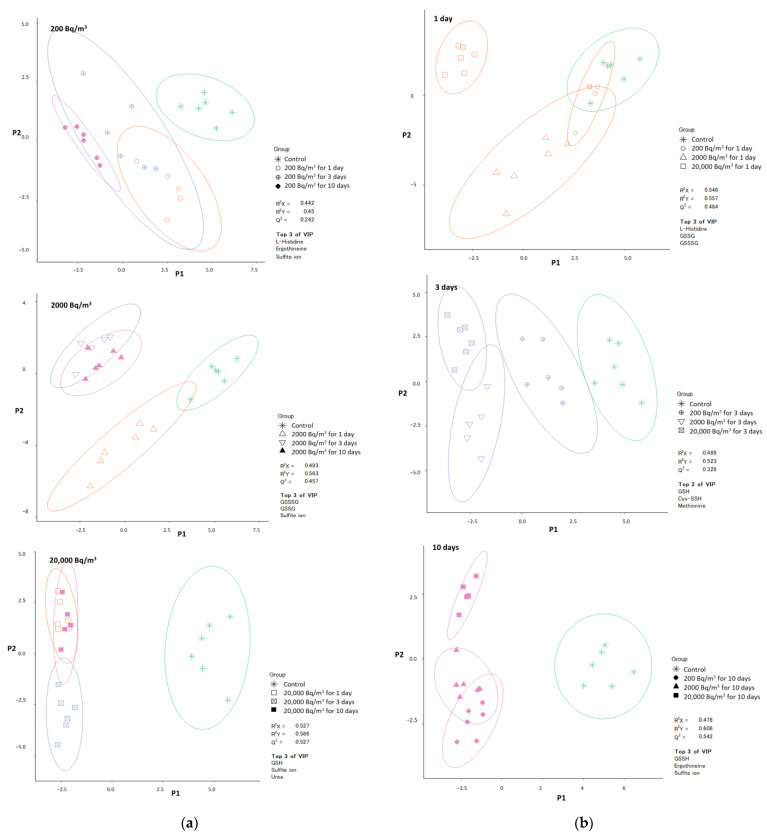
Clustering by PLS-DA. PLS-DA was performed by (**a**) radon concentration or (**b**) radon inhalation time.

We divided the data into radon concentration (Figure 7a) and radon inhalation time (Figure 7b), and performed PLS-DA to characterize the 27 detected metabolites following radon inhalation. Although the accuracy was not enough for the classification, it gave a rough estimate of the changes in metabolites through radon inhalation. Only the top three VIP values are shown as important factors, and the circles indicate the 95% confidence interval (Figure 7). Classifying the data by the groups was not easy. The circle of the control group overlapped that of the 200 Bq/m^3^ group for the 1-day group when we tried analyzing only the 1-day groups (Figure 7b). However, there was a difference between them if we tried analyzing only the 200 Bq/m^3^ groups (Figure 7a). The results in Figure 7a,b are different because the target analytical data were different. The control and the 200 Bq/m^3^ group for the 1-day group were classified when the target analytical data were rational. Furthermore, we could not identify the radon exposure amount because some circles of the radon exposed group overlapped each other. For instance, the groups of the 2000 Bq/m^3^ groups for 3 and 10 days and the 20,000 Bq/m^3^ groups for 1 and 10 days were particularly difficult to understand.

**Figure 8:**
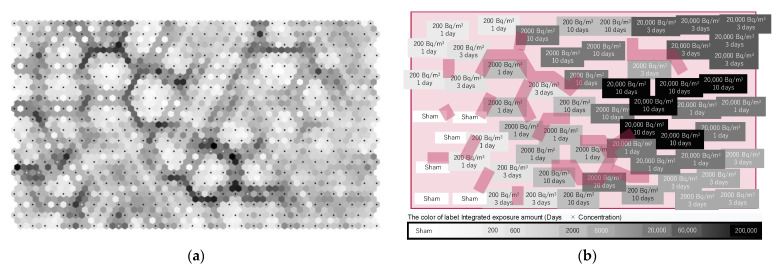
Feature extraction by SOM. (**a**) There were 600 hexagonal units, which had the data of the 27 detected metabolites on the output map. Units with similar patterns were placed into similar locations and a darker color unit was more different from the neighboring nodes. (**b**) The borderline and best matching units each with 60 input data (six mice per group in 10 groups) are shown on the conceptual diagram of the output map.

We represented many units that reflected the data of the metabolomics analysis on the output map using ML-SOM to understand the effects of radon inhalation (Figure 8a). SOM places data with similar patterns into similar locations (unit). The output map was drawn using gray-scale; the characteristics of the light color unit were similar to that of the neighboring units and the characteristics of the darker color node were more different from those of the neighboring units. In other words, the darker line depicted the difference in the characteristics of the units on the output map. Figure 8b is the conceptual diagram of Figure 8a. The borderlines, which show the differences in each unit, are depicted on the conceptual diagram. Sixty units (six mice per group in 10 groups) were selected as the best matching units with input data from 600 (30 × 20) units, and they were labeled in the output map. This output map shows metabolite content changes dependent on radon exposure conditions from the lower left to the upper right, roughly. However, we found that the 1-day groups were closer to the 10-day groups than the 3-day groups in the groups of more than 200 Bq/m^3^. Moreover, classifying the data by radon concentration seemed difficult in the 10-day groups. Therefore, the higher the radon concentration or the longer the radon exposure time, the less the changes in metabolites related to the radon exposure amount.

## 4. Discussion

We previously reported that various diseases are suppressed because of the increased antioxidative function by radon inhalation in mice [9,10,11,12,13]. The present study focused on metabolites containing sulfur such as G-SH in the mouse brain, because radon inhalation (2000 Bq/m^3^ of radon for 24 h) increased the amounts of antioxidants (such as G-SH) in the brains and had a protective antioxidative effect against transient global cerebral ischemic injury [10]. We measured the associated metabolites to (1) investigate whether there is evidence of a clear indication of radon exposure by metabolomics analysis, (2) discuss the effects of radon inhalation on G-SH- and Cys-SH-related substances, and (3) comprehensively explain the characteristics of the output that was produced by visualizing multiple raw data using ML.

We exhaustively investigated the changes in sulfur-related metabolites in mouse brains following radon inhalation at background levels of 200, 2000, or 20,000 Bq/m^3^ for 1, 3, or 10 days. We detected 27 metabolites in the mouse brains (Table 1). We found that the L-methionine, urea, G-SH, and sulfite ions responded to all radon exposure conditions (Figure 1 and Figure 2). Moreover, Cysteinylglycine, Ergothioneine, L-cystathionine, O-acetylserine, Methylcysteine, S-adenosylhomocysteine, Cys-S-SH, G-SH, and Thiosulfate ions were significantly correlated with radon inhalation days or radon concentration (Table 2). The result of PLS-DA showed that the control group was different from the radon exposure group (Figure 7). The factors in the top three VIP values, such as L-histidine, Ergothioneine, sulfite ion, GS-S-SG, GS-SG, G-SH, urea, Cys-S-SH, L-methionine, and G-S-SH, were important for the radon exposure effect assessment. Although the theoretical background of the VIP score of PLS-DA is lacking [28], these substances are typical oxidative stress-related metabolites [29]. Thus, our results show that these metabolites could clearly indicate radon exposure, because these were highly reactive metabolites to radon exposure. In particular, G-SH was also observed as an important metabolite in all data analysis. Further studies are needed to investigate the G-SH-related substances in detail.

**Figure 9:**
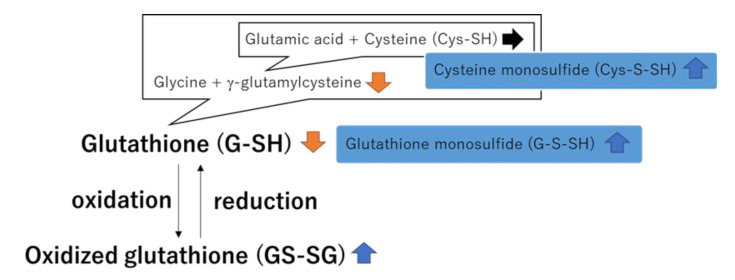
Changes in G-SH-, Cys-SH-, and RSS-related metabolites after radon inhalation. The figure shows an illustration of the reduction and oxidization of G-SH. G-SH is synthesized from γ-glutamylcysteine and glycine. γ-glutamylcysteine is synthesized from glutamic acid and Cys-SH. The sulfur ion possibly chemically binds with G-SH or Cys-SH. The changes in G-SH- and Cys-SH-related metabolites are indicated by arrows. 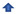 indicates an increase, 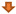 indicates a decrease, and 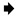 indicates no change in radon exposure groups compared with the control.

Sulfur is an essential element for living organisms, and reactive sulfur species (RSS) exists in all organs at appreciable concentrations [7,29,30,31]. Zhang et al. demonstrated that RSS and related molecules have anti-inflammatory properties (e.g., Cys-S-SH has an anti-inflammatory effect) [8,32]. Many studies have also indicated the importance of RSS in redox regulation and electrophilic signaling. However, to the best of our knowledge, no study has focused on the effect of RSS after irradiation. According to the physiological-based pharmacokinetic modeling of radon inhaled, the absorbed dose in the mouse brains per unit of ambient radon concentration and unit radon inhalation time ranged from 0.047 to 0.075 nGy/(Bq/m^3^)/day in the present study [33]. It is expected that mouse brains were exposed to radiation at approximately 9.4 nGy (the minimum dose was 200 Bq/m^3^ for 1 day) to 15.0 μGy (the maximum dose was 20,000 Bq/m^3^ for 10 days) in this study. It is important to note that the biological effects were observed in even lower doses than 15.0 μGy. The G-SH/GS-SG ratio, a typical marker of oxidative stress, changed following radon inhalation (Figure 4). Meanwhile, we found an observable increase in RSS, such as G-S-SH and Cys-S-SH, after radon inhalation (Figure 2 and Figure 9). In addition, radon inhalation had a protective antioxidative effect against transient global cerebral ischemic injury [10]. Therefore, we considered that RSS could produce a biological defense against the imbalance of the redox state at very low-dose irradiation following radon inhalation. We intend to show how RSS is involved in the alleviation of symptoms of various diseases by radon inhalation in the future.

We considered that the labels on the output map could be arranged according to the radon exposure amount, because we have already demonstrated the availability of data analysis using SOM [34] (Figure 8). In previous studies, we presented evidence of the effectiveness of the treatments of oxidative-stress-related disorders by radon inhalation at thousands of Bq/m^3^ for 1 day [9,10,11,12,13]. In Figure 8, the borderlines are represented around the units, which show the data for radon inhalation at 2000 Bq/m^3^ for 1 day. We assume that this radon exposure condition has plenty of meaning for treatment through radon inhalation. The visualization by ML enabled us to uncover valuable information that is consistent with our previous reports. Moreover, although we understood easily how the groups of sham and of 200 Bq/m^3^ for 1 day were similar, the inconsistent results in the case of the higher concentrations or the longer inhalation times were remarkable in the simple regression analysis, PLS-DA, and SOM (Figure 5, Figure 6, Figure 7 and Figure 8). Therefore, this result showed the health effects of radon, especially for the elucidation of the therapeutic effect mechanism of using radon. Another discussion regarding the larger exposure conditions needs to occur separately. In the future, this will lead us to discuss the issue the dose rate effect, risks/benefits of irradiation, and so on.

## 5. Conclusions

We revealed the biological effects of low-dose irradiation, focusing on the sulfur-related metabolites in mouse brains following radon inhalation. The sulfur metabolism was certainly related to low-dose irradiation after radon inhalation (e.g., the L-methionine, urea, G-SH, and sulfite ion responded to all radon exposure conditions). Moreover, radon inhalation increased G-S-SH and Cys-S-SH under the unbalanced redox state. However, the changes in metabolites due to radon were not completely dose-dependent. Our findings suggest that the living body regulates oxidative stress following radon inhalation. Although determining the health risks and benefits of radon is not easy, we showed the state of sulfur-related metabolites in mouse brains under the exposure condition for radon therapy.

## Figures and Tables

**Table 1:** List of 27 metabolites detected in the mouse brains.

Metabolites		
Adenosine 3′,5′-diphosphate	L-Histidine	O-Acetylserine
Cysteine monosulfide (Cys-S-SH)	L-Homoserine	S-Adenosylhomocysteine
Cysteinylglycine	L-Lactic acid	S-Adenosylmethionine
Ergothioneine	L-Methionine	Sulfide ion
Glutathione (G-SH)	L-Serine	Sulfite ion
Glutathione monosulfide (G-S-SH)	Methylcysteine	Taurine
γ-Glutamylcysteine	Oxidized glutathione (GS-SG)	Thiamine
L-Cystathionine	Oxidized glutathione disulfide (GS-SS-SG)	Thiosulfate ion
L-Cysteine (Cys-SH)	Oxidized glutathione monosulfide (GS-S-SG)	Urea

**Table 2:** Significant correlation coefficient with radon inhalation days and radon concentration (Bq/m^3^). (**a**) Correlation coefficient with radon inhalation days. (**b**) Correlation coefficient with radon concentration.

(a)		
Metabolites	Correlation Coefficient	
Ergothioneine	0.65	***
Cycteinylglycine	0.56	***
(b)		
Metabolites	Correlation Coefficient	
Cysteine monosulfide	0.77	***
S-Adenosylhomosyvteine	0.55	***
O-Acetylserine	0.53	**
Methylcysteine	0.48	*
Thiosulfate ion	−0.53	**
Glutathione	−0.56	***
L-Cystathionine	−0.59	***

Note: The 27 detected metabolites and radon inhalation time (days) or radon concentration (Bq/m^3^) were analyzed using a Pearson product–moment correlation coefficient. Significantly correlated metabolites with (a) inhalation days or (b) radon concentration. * *p* < 0.05, ** *p* < 0.01, *** *p* < 0.001.

## Data Availability

Not applicable.
